# Long QT syndrome and left ventricular non-compaction in a family with KCNH2 mutation: A case report

**DOI:** 10.3389/fped.2022.970240

**Published:** 2022-08-04

**Authors:** Thomas Caiffa, Antimo Tessitore, Loira Leoni, Elena Reffo, Daniela Chicco, Biancamaria D'Agata Mottolese, Elisa Rubinato, Giorgia Girotto, Stefania Lenarduzzi, Egidio Barbi, Marco Bobbo, Giovanni Di Salvo

**Affiliations:** ^1^Department of Paediatrics, Institute for Maternal and Child Health IRCCS ‘Burlo Garofolo', Trieste, Italy; ^2^Department of Medicine, Surgery and Health Sciences, Department of Paediatrics, University of Trieste, Trieste, Italy; ^3^Cardiology Clinic, Department of Cardiac, Thoracic, Vascular Sciences and Public Health, University of Padova Medical School, Padova, Italy; ^4^Pediatric Cardiology Unit, Department of Woman and Child's Health, University of Padova Medical School, Padova, Italy; ^5^Medical Genetics, Institute for Maternal and Child Health – IRCCS “Burlo Garofolo,” Trieste, Italy; ^6^Department of Medicine, Surgery and Health Sciences, University of Trieste, Trieste, Italy

**Keywords:** KCNH2 variant, left ventricular non-compaction, long QT syndrome, LQTS, LVNC

## Abstract

**Background:**

Left ventricular non-compaction (LVNC) is an abnormality of the myocardium, characterized by prominent left ventricular trabeculae and deep inter-trabecular recesses. Long QT syndrome (LQTS) is a cardiac ion channelopathy presenting with a prolonged QT interval on resting electrocardiogram and is associated with increased susceptibility to sudden death. The association between LVNC and LQTS is uncommon.

**Case presentation:**

We report an Italian family with a novel pathogenic KCNH2 variant who presented with clinical features of LVNC and LQTS. The proband came to our attention after two syncopal episodes without prodromal symptoms. His ECG showed QTc prolongation and deep T wave inversion in anterior leads, and the echocardiogram fulfilled LVNC criteria. After that, also his sister was found to have LQTS and LVNC, while his father only presented LQTS.

**Conclusions:**

Physicians should be aware of the possible association between LVNC and LQTS. Even if this association is rare, patients with LVNC should be investigated for LQTS to prevent possible severe or even life-threatening arrhythmic episodes.

## Background

The association of long QT syndrome (LQTS) and left ventricular non-compaction (LVNC) is known but relatively uncommon. In 2017, the first study reported data from four family members across two generations with evidence of QTc interval prolongation and LVNC associated with a pathogenic variant in KCNQ1 (1). Another report described the case of a 5 years-old girl with LVNC and prolonged QTc after an episode of aborted sudden death. The genetic analysis showed evidence of a previously reported pathogenic KCNQ1 variant: c1831G>T, D611Y (2). Finally, two other patients with LQTS and LVNC were described, with genetic variants in KCNH2: A561V and D501N (3).

We present the case of a family with evidence of LQTS and LVNC associated with a pathogenic variant in KCNH2.

## Case presentation

### Case 1

**Figure 1:**
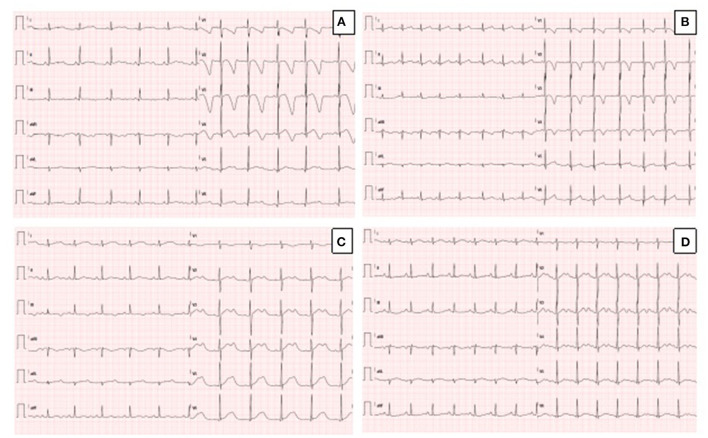
**(A)** Resting ECG in the proband, showing sinus rhythm, QTc prolongation (567 msec) and deep T wave inversion in anterior leads. **(B)** Resting ECG in the sister of the proband, showing sinus rhythm, normal QTc (442 msec) and T wave inversion in anterior leads. **(C)** Resting ECG in the father of the proband during mild hypokalemia (K+ 3.3 mEq/L), showing sinus rhythm, prolongation of QTc (614 msec) and biphasic T-waves. **(D)** Resting ECG in the father of the proband during normokalaemia (K+ 4.1 mEq/L), showing sinus rhythm, prolongation of QTc (550 msec), and biphasic T-waves (pseudo-U waves).

**Figure 2:**
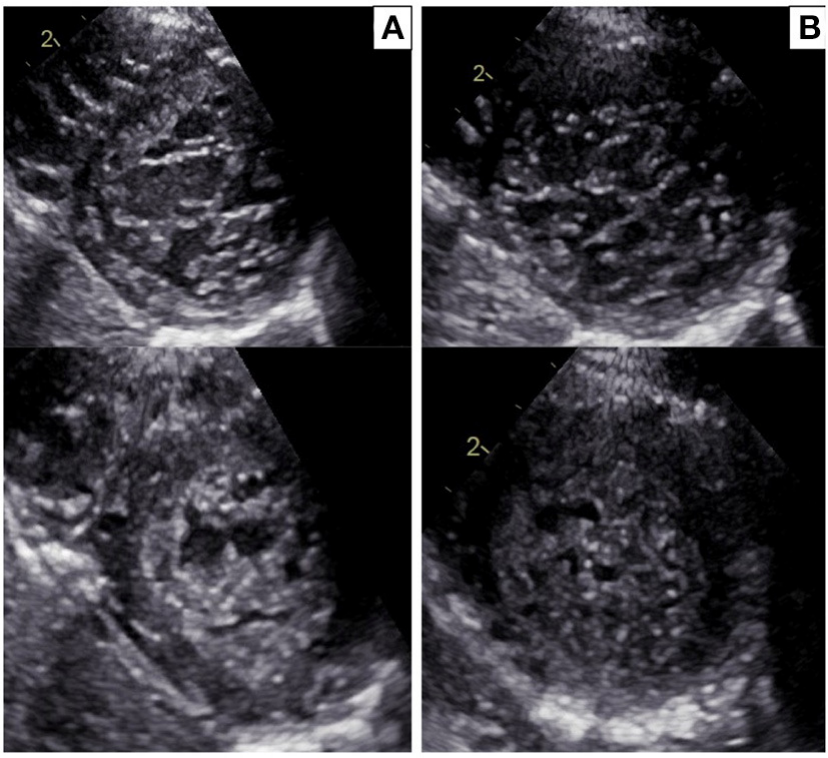
**(A)** Echocardiographic parasternal short-axis view in the proband: diastole (upper panel) and systole (bottom panel). Trabecular/compacted end-systolic wall thickness ratio >2 (positive Jenni criteria for LVNC). **(B)** Echocardiographic parasternal short-axis view in the sister of the proband: diastole (upper panel) and systole (bottom panel). Trabecular/compacted end-systolic wall thickness ratio >2 (positive Jenni criteria for LVNC).

**Figure 3:**
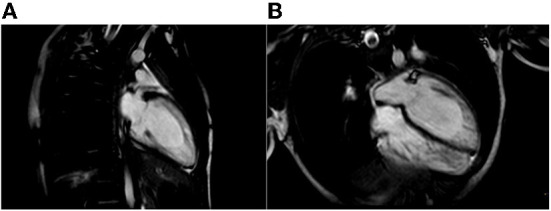
Cardiac Magnetic Resonance end-diastolic 2-chamber **(A)** and 4-chamber **(B)** SSFP cine view showing an increased trabecular/compacted wall thickness ratio.

The proband was a 5-year-old boy with no known family history of heart disease and sudden cardiac death (SCD) at initial evaluation. We first met the patient after a syncopal episode when he bent down to collect his eyeglasses from the floor. The episode was self-resolving, with the recovery of consciousness in 1–2 min. The electrocardiogram showed sinus bradycardia, long QTc interval (567 ms) and deep inverted/biphasic T-waves in anterior leads V1–V4 (Figure 1A). Laboratory tests including electrolytes, haemochrome, high sensibility troponin I, reactive protein C and creatine phosphokinase were normal. An echocardiogram was performed, which fulfilled Jenni's criteria for LVNC (Figure 2A). Cardiac magnetic resonance end-diastolic 2-chamber and 4-chamber SSFP cine view showed an increased trabecular/compacted wall thickness ratio, confirming the echocardiographic finding (Figure 3).

**Figure 4:**
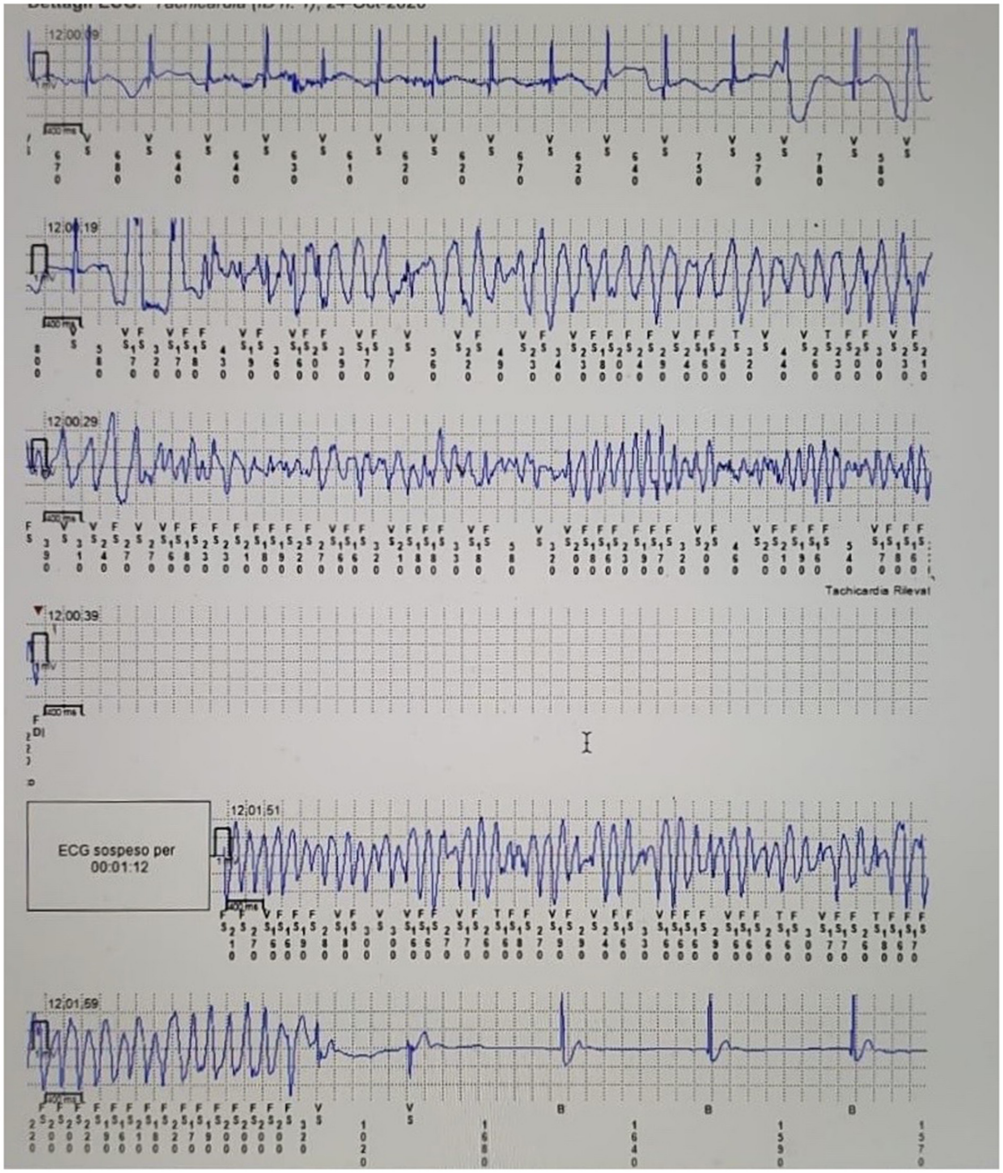
Proband's loop Recorder trace during cardiac arrest, showing QTc prolongation, ectopic ventricular beats and ventricular fibrillation.

Biventricular dimensions, wall thickness, and systolic and diastolic functions were regular. ECG-Holter monitoring proved the prolongation of QTc interval, reporting value between 540 and 570 msec, with no evidence of ventricular arrhythmia, short phases of isorhythmic dissociation and sinus bradycardia and no evidence of ventricular arrhythmia. An internal loop recorder was implanted, and blood samples for genetic testing were collected. 1 month after discharge, the patient had another syncopal episode after auditory stimulation by the doorbell during complete well-being. The event resolved spontaneously in 2 min. The analysis of the loop recorder revealed an episode of ventricular fibrillation that lasted about 1.40 min and was preceded by a brief phase of ventricular bigeminy (Figure 4). The patient was urgently admitted to the hospital and administrated antiarrhythmic therapy with intravenous magnesium. In the following days, therapy with nadolol 1 mg/kg/die and mexiletine 6.6 mg/kg/die was initiated and titrated. Moreover, an implantable cardioverter-defibrillator (ICD) was implanted for secondary prevention. A few weeks later, an NGS panel performed in the proband DNA, analyzing 16 genes related to LQTS, detected a pathogenic heterozygous variant c.1889T > G p. (V630G) in the KCNH2 gene (NM_000238.3). The missense variant is predicted as damaging by the *in silico* tools used during data analysis (SIFT, Polyphen2_HVAR, DANN, Mutation Taster, Mut Pred), and it is classified as pathogenic by ACMG classification. 1 year later, the child is still well-being, and no major arrhythmic events have occurred.

### Case 2

The 3-year old sister of the proband was evaluated 1 week after the first syncopal episode of her brother. Her medical history was unremarkable, with no syncope or other cardiac symptoms. The ECG showed sinus rhythm, QTc interval 442 ms and T wave inversion in the anterior leads V1-V4 (Figure 1B). Except for the duration, the QTc and ECG of the girl and her brother looked very similar, and an echocardiogram proved normal biventricular function and criteria for apical LVNC (Figure 2B). ECG-Holter monitoring confirmed standard sinus rhythm, average QTc values, and no episodes of bradycardia or hyperkinetic arrhythmias. Blood samples for genetic testing were collected, which led to the same pathogenic variant of KCNH2 identified in the proband. Beta-blocker therapy with nadolol 1 mg/kg/die was started.

### Case 3

We studied the proband's parents. The mother's ECG and echocardiogram, both previously performed at our center, were average, while the father's ECG, although not available, was reported as standard. The paternal grandfather died in a car accident at the age of 35.

A few days after the clinical evaluation of the proband's sister (case 2), the father was admitted for a minor pulmonary embolism. The analysis of the clinical documentation showed that he had a transient prolongation of the QTc interval (614 ms) during mild hypokalemia (K+ 3.3 mEq/L), Figure 1C.

The man had no history of syncope. We revised his previous ECGs that indicated a wide range of T wave alternans, biphasic T waves (often described as U waves) and a long T wave terminal slope (Figure 1D). We performed whole exome sequencing analysis (WES) on the father DNA, and the test revealed the same pathogenic variant of KCNH2, c.1889T>G p. (V630G) (see Sanger sequencing on Supplementary Material 1). The echocardiography performed during the hospitalization for pulmonary embolism was standard. Holter-ECG revealed frequent ventricular ectopic beats (4,500/24 h) that disappeared during the exercise test physical activity. The QTc was prolonged and did not significantly modify during the stress test at 1 and 4 min after recovery. The patient was advised to start therapy with nadolol. Supplementary Material 2 illustrates the family pedigree.

## Discussion and conclusions

Initially ascribed to a consequence of arrested cardiac morphogenesis (4), LVNC is a morphological abnormality of the myocardium, characterized by prominent left ventricular trabeculae and deep inter-trabecular recesses (5). It occurs as a genetic trait and is often associated with congenital heart defects and different cardiomyopathy phenotypes. The term non-compaction refers to an abnormal ventricular morphology characterized by the coincidence of a thin outer layer of regular or ‘compacted' myocardiumù with an inner ‘non-compacted' layer consisting of prominent muscular trabeculae and intra-trabecular recesses that communicate with the ventricular cavity. Non-compaction entails a premature arrest of the myocardium development that results in the persistence of an abnormal trabecular architecture. As cardiomyocytes are terminally differentiated cells, the new trabecular formation cannot arise in adults' hearts, but increased ventricular mass can occur due to hypertrophy of existing cardiomyocytes (6). LVNC is recognized in clinical practice with a reported prevalence of 1:5,000 individuals in the general population and 3–4% of adults with heart failure (7); even if the LVNC prevalence varies according to the imaging modality, the diagnostic criteria and the study population. Abnormal cardiac mechanics affecting apical rotation are a distinctive feature of LVNC in children (8). Among the variants in 66 genes, MYH7 is the most frequently implicated in the LVNC development, observed in one-quarter of patients.

Additionally, some genes lead to complex and critical clinical disease syndrome associated with LVNC, such as Rubinstein–Taybi syndrome (i.e., ABCC9 gene), Ehlers–Danlos syndrome (i.e., COL3A1 gene), Sotos syndrome (i.e., NSD1 gene), and LEOPARD syndrome (i.e., PTPN11 gene) (9). Patients with LVNC risk developing heart failure, atrial and ventricular arrhythmias, and systemic embolic events in pediatric and adult populations (10). Diagnosis is usually confirmed by cardiac MRI, looking for a specific ratio of non-compacted to compacted myocardium (11, 12). However, it can be a challenging diagnosis since trabeculation can occur as a physiological trait, especially in athletes whose increased left ventricle preload and afterload can determine an augmented trabeculation (13).

LQTS is a cardiac ion channelopathy characterized by a prolonged QT interval on an electrocardiogram associated with increased susceptibility to torsade de pointes and sudden death (14). Channelopathies cause up to 1% of sudden cardiac death. Congenital and acquired LQTS is caused by genetic or drug-induced perturbations of critical ion channels that generally ensure the proper functioning of the beating heart (15). Regarding congenital forms, approximately 75% of patients with LQTS have a mutation in one of the three significant LQTS genes as follows: KCNQ1-encoded I_Ks_ potassium channel (LQT1), KCNH2-encoded I_Kr_ potassium channel (LQT2), and SCN5A-encoded I_Na_ sodium channel (LQT3) (16).

The association between LVNC and LQTS is rare. Although the mechanism is unclear, several hypotheses have been proposed, such as the direct protein-protein interaction of the LQTS gene with the sarcomere or that LVNC is an adaptive remodeling feature in response to impaired conduction (10). Notably, subclinical cardiomyopathic changes were reported in 20% of patients in one study (17). Another investigation demonstrated subtly reduced systolic and diastolic function in patients with LQTS than in controls, further suggesting that LQTS may not purely be an electrical disease (18). This evidence implies that, while directly causing the long QT syndrome, the mutation may also play a role in the LVNC development. Teng et al. (19) pointed out that ion channels are critically involved in the development of vascular smooth muscle since it is demonstrated that homozygous deletion of KCNH2 can lead to cardiac developmental defects that finally result in embryonic lethality.

Some reports have already described the association between LVNC and LQTS. Xu et al. (20) reported a family in which a KCNH2 missense mutation (c.818 C > T, p.T273M; NM_001204798) located in the pore area of the hERG channel, a pathogenetic loss-of-function mutation, caused long QT among the proband, her younger sister and her mother with a significant non-compaction of the myocardium in the proband and her mother. Ogawa et al. (3) reported two cases of LVNC with LQTS, the first one with KCNH2 missense mutation (A561V) while the second one with the mutant KCNH2 (D591N). Apart from KCNH2, other LQTS variants were found in genes not previously reported in association with LVNC, including KCNE1, KCNQ1, and KCNJ2 (10).

We identified a novel pathogenic variant in the KCNH2 gene, c.1889T > G p. (V630G), associated with reasonable certainty with both LVNC and LQTS2, as the negative father's exome, the predicted pathogenetic variant on a gene known for the cardiac phenotype in question and the family segregation are suggestive of the association. Anyway, further investigations are needed to confirm this association since gene variants linked to LVNC were not tested in the proband and his sister.

The proband and his sister presented deep, inverted/biphasic T-waves in anterior leads V1–V4, but only the proband and his father had an extremely long QTc interval. ECG-Holter monitoring of the proband confirmed the prolongation of QTc interval, with no evidence of ventricular arrhythmia, while ECG-Holter monitoring of his father showed frequent ventricular ectopic beats (4,500/24 h) disappearing during exercise test physical activity. Conversely, ECG-Holter monitoring of his sister was regular. In addition, the proband and his sister fulfilled the LVNC criteria, restricted to the apex for his sister, with a normal biventricular function. On the contrary, LVNC was absent in proband's father. All the cases started treatment, and an ICD was implanted in the proband.

These reports further suggest that physicians should know the possible association between LVNC and LQTS. Even if this association is rare, patients with LVNC should be investigated for LQTS to prevent possible severe or even life-threatening arrhythmic episodes. Moreover, family members of a proband with LQTS and/or LVNC should always receive cardiological screening and follow-up, considering the possibility of phenotypic variability and different age onset.

## Data availability statement

The original contributions presented in the study are included in the article/supplementary material, further inquiries can be directed to the corresponding author/s.

## Ethics statement

Written informed consent was obtained from the minor(s)' legal guardian/next of kin for the publication of any potentially identifiable images or data included in this article.

## Author contributions

AT and TC wrote the first draft of the manuscript. MB, TC, DC, LL, and ER saw the patient clinically and revised the final manuscript. ER, GG, and SL performed the whole exome sequencing on the DNA and the genetic consultation. EB, BD'A, and GD critically revised the manuscript for relevant intellectual content. All authors read and approved the final manuscript.

## Funding

This work was supported by the Ministry of Health, Rome - Italy, in collaboration with the Institute for Maternal and Child Health IRCCS Burlo Garofolo, Trieste – Italy.

## Conflict of interest

The authors declare that the research was conducted in the absence of any commercial or financial relationships that could be construed as a potential conflict of interest.

## Publisher's note

All claims expressed in this article are solely those of the authors and do not necessarily represent those of their affiliated organizations, or those of the publisher, the editors and the reviewers. Any product that may be evaluated in this article, or claim that may be made by its manufacturer, is not guaranteed or endorsed by the publisher.

## Abbreviations

ECG: Electrocardiogram
ICD: Implantable cardioverter-defibrillator
LQTS: Long QT syndrome
LVNC: Left ventricular non-compaction
SCD: Sudden cardiac death.
