# Supplementary material for: Long QT syndrome and left ventricular non-compaction in a family with KCNH2 mutation: A case report
Source: Front Pediatr. 2022 Aug 4;10:970240. doi: 10.3389/fped.2022.970240 (PMC9386155; doi:10.3389/fped.2022.970240)

KCNH2 (NM_000238.3) c.1889T>G p.V630G

>chr7:150648460+150648653
194bp
TAGCAGCCTCAGTTTCCTCC
AAGTATGTGACGGCGCTCTA

TAGCAGCCTCAGTTTCCTCCaacttgggttcctccaccgtgggctctccc
cgccgcccgcccctgggcacactcacagccaatgagcatgacgcagatgg
agaagatcttctctgagttggtgttgggagagacgttgccgaagcccaca
ctggtgaggctgctgaaggtgaagTAGAGCGCCGTCACATACTT

CTCACCAGTGTGGGCTTCGGCAACG T CTCTCCCAACACCAACTCAGAGAAG
CTCACCAGTGTGGGCTTCGGCAACG G CTCTCCCAACACCAACTCAGAGAAG

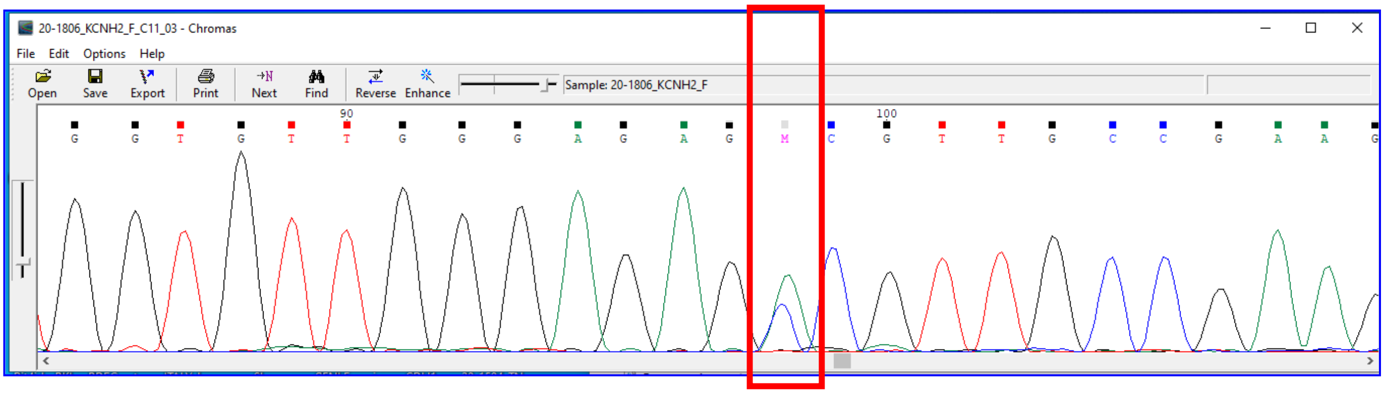

Supplement: Supplementary file 1 [file Data_Sheet_1.PDF]
