# Supplementary figures and images for: Long QT syndrome and left ventricular non-compaction in a family with KCNH2 mutation: A case report
Source: Front Pediatr. 2022 Aug 4;10:970240. doi: 10.3389/fped.2022.970240 (PMC9386155; doi:10.3389/fped.2022.970240)

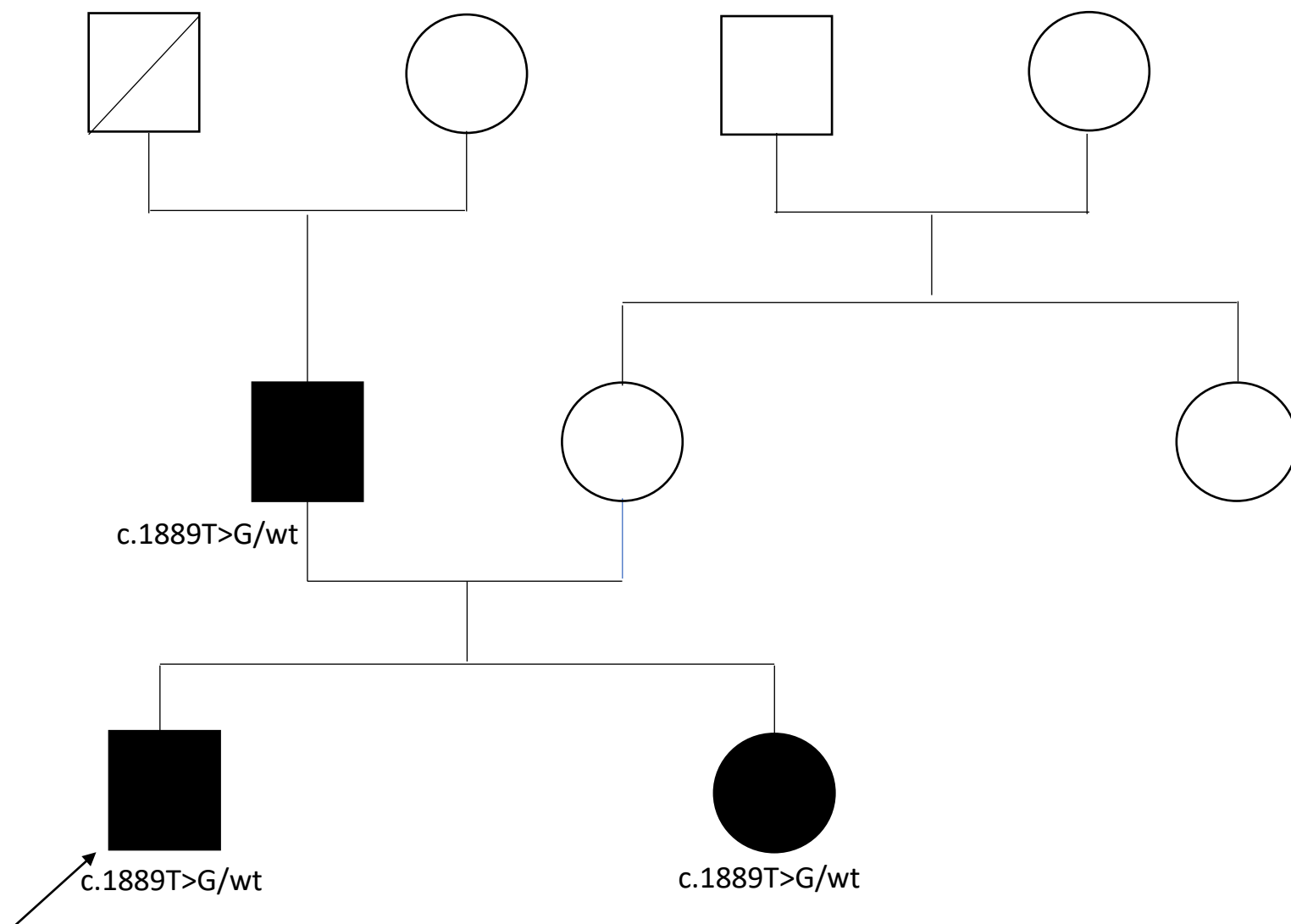

Supplement: Supplementary file 2 [file Data_Sheet_2.PDF]
